# Supplementary figures and images for: First known trace fossil of a nesting iguana (Pleistocene), The Bahamas
Source: PLoS One. 2020 Dec 9;15(12):e0242935. doi: 10.1371/journal.pone.0242935 (PMC7725343; doi:10.1371/journal.pone.0242935)

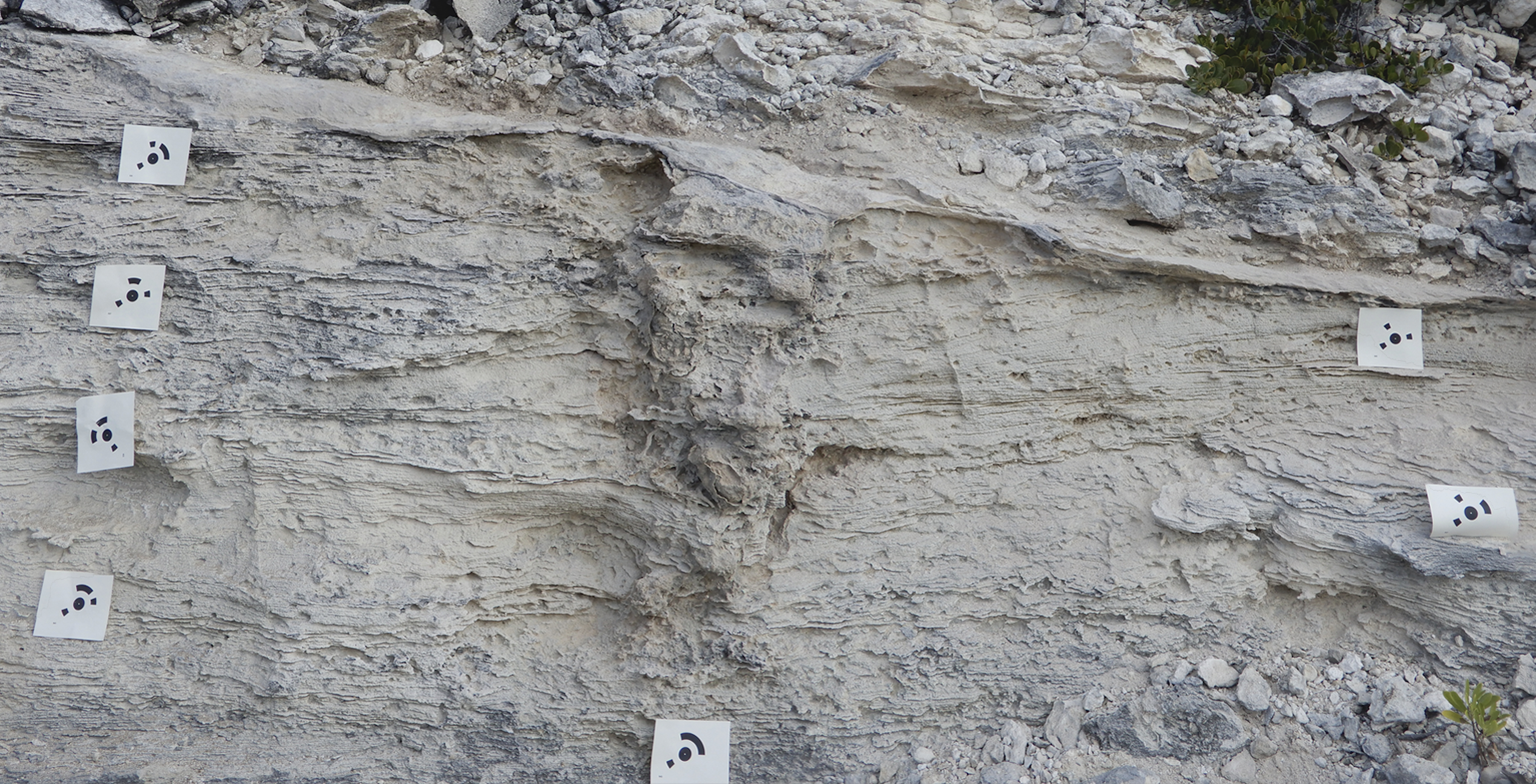

Supplement: S1 Fig — (TIF) [file pone.0242935.s001.tif]
