# Supplementary material for: First known trace fossil of a nesting iguana (Pleistocene), The Bahamas
Source: PLoS One. 2020 Dec 9;15(12):e0242935. doi: 10.1371/journal.pone.0242935 (PMC7725343; doi:10.1371/journal.pone.0242935)
Supplement: S1 File — (DOCX) [file pone.0242935.s002.docx]

Martin et al., *First Known Trace Fossil of a Nesting Iguana (Pleistocene), The Bahamas*

**Supporting Figures, Files**

**Supporting Figure 1.** Photogrammetry markers around trace fossil of interpreted iguana nesting burrow, placed at approximately 20-cm intervals and about 1 m left and right of central part of the structure and below. Figure is composite from five overlapping digital photos taken on March 15, 2018; composite made using Photomerge function in Adobe Photoshop™, Version 12.0.

**Supporting Figure 2.** Link to [preliminary photogrammetric model](http://geoam.ecdsdev.org/unity/) (Unity WebGL build) of fossil iguana burrow on San Salvador Island (The Bahamas), rendered using AgiSoft Metashape™ photogrammetry software. Current model uses accelerated solar pathway (dawn to dusk) matching the latitude-longitude of the trace fossil locality. Original model is in millions of polygons and hence could not be displayed here without causing user problems, but will be optimized later for viewing online.

Link: <http://geoam.ecdsdev.org/unity/>

**Supporting Figures 3-4**. Link to two videos (.mov files) of photogrammetric model of fossil iguana burrow on San Salvador Island (The Bahamas), based on Unity WebGL build in Supporting Figure2. Video 1 is with counterclockwise rotation and video 2 with clockwise rotation, each with dawn to dusk lighting matching the latitude-longitude of the trace fossil locality.

Link: <http://geoam.ecdsdev.org/videos/>

**Supporting Files 1-2.** Links to folders containing original photographs used for photogrammetry model of fossil-iguana burrow (Supporting Figure 2), San Salvador Island, The Bahamas: [File 1](http://geoam.ecdsdev.org/photos1/) (n = 50), [File 2](http://geoam.ecdsdev.org/photos2/) (n = 24). Photographs taken with hand-held Nikon CoolPix A900 digital camera on March 15, 2018.

Link 1: <http://geoam.ecdsdev.org/photos1/>

Link 2: <http://geoam.ecdsdev.org/photos2/>
